# Comparison of Latest and Innovative Silica-Based Consolidants for Volcanic Stones

**DOI:** 10.3390/ma14102513

**Published:** 2021-05-12

**Authors:** Abner Colella, Ilaria Capasso, Fabio Iucolano

**Affiliations:** 1 Dipartimento di Scienze della Terra, dell’Ambiente e delle Risorse(DiSTAR), University of Naples Federico II, Via Vicinale Cupa Cintia 21, 80126 Naples, Italy; abner.colella@unina.it; 2 Department of Engineering and Geology, University of Chieti-Pescara “G d’Annunzio”, Viale Pindaro 42, 65122 Pescara, Italy; 3 ACLabs-Applied Chemistry Labs, Department of Chemical, Materials and Industrial Production Engineering, University of Naples Federico II, P.le Tecchio 80, 80125 Naples, Italy; iucolano@unina.it

**Keywords:** lithium silicate, nano-silica, inorganic stone consolidants, Neapolitan Yellow Tuff, Campanian Ignimbrite

## Abstract

This research explores the new perspectives in conservation and protection of two macroporous tuff stones, widely employed in the architectural heritage of Campania region, characterized by highly heterogeneous rock fabric and texture and a variable mineralogical composition that represent crucial factors responsible for their weak durability. The consolidation treatments were performed with a recently and widely used suspension of nano-silica crystals in water and with a lithium silicate solution that has received up to now scarce attention as a consolidant agent. Physical investigations (open porosity, Hg porosimetry, water absorption), morphological observations (SEM analyses) and visual appearance test (colorimetric measurements), along with assessments of performance indicators such as ultrasonic pulse velocity, surface cohesion test (peeling test) and durability test (salt crystallization), were carried out to investigate the consolidation effectiveness. Overall, lithium silicate consolidant showed a better behavior in terms of superficial cohesion, a most successful strengthening action and a considerable enhancement of salt resistance.

## 1. Introduction

The progressive and inevitable deterioration process of stone heritage and buildings went through a significant acceleration over the last century and is even expected to grow at a higher rate in the near future due to, among other things, increasing air pollution [1,2,3,4,5,6,7,8] and enhanced deterioration due to deposition of soluble salts and/or aerosols or reactions of the stone with atmospheric pollutants, also associated with climate change [9,10,11,12,13,14]. So, in the last decades, the increasing concern over the degradation of the worldwide cultural heritage has motivated researchers to find ever more innovative and effective solutions to preserve the integrity of the historical patrimony and at the same time to guarantee its continuing fruition [15,16,17].

In general, only the complete knowledge of the specific mechanisms responsible for the stone degradation, whether they are intrinsic properties of the stone (mineral composition, textural characteristics and pore/capillary structure, etc.) or extrinsic factors of decay (microclimatic conditions such as temperature and humidity changes, water/moisture transportation, air pollution, biological activities, etc.), enable planning the most appropriate strategies needed to reduce weathering of stone [18,19,20]. The conservation of historic and culturally relevant stone artworks and buildings involves the on-site protection and/or restoration, usually achieved by re-establishing grain-to-grain cohesion of damaged stone through the application of organic polymers, alkoxysilanes or inorganic consolidant compounds [15,21,22]. Moreover, one of the main purposes of consolidation treatments that cannot be overlooked is to avoid or reduce the water/moisture penetration in stones, in order to minimize the rate of stone decay due to freeze–thaw cycles inside the pore pattern or the intraporous crystallization of soluble salts transferred by the water [9,23,24,25]. The improvement of the water repellence properties of deteriorated stone substrates is generally achieved using polymeric films to promote the reduction in the surface tension of the substrates [26,27,28], but the use of organic polymers presents several limitations and inconveniences, especially in terms of physical–chemical incompatibility. In addition, the same formation of polymeric protective films might cause further damage because of pore blocking, which negatively affects the water vapor transport mechanisms [15,29]. On the other hand, inorganic consolidants, especially silicon-based compounds, have been extensively taken into consideration in the last years, especially because they offer a remarkable opportunity to design consolidants with higher compatibility with the original stone substrates [30,31,32]. Consolidants are often alkoxysilane products, so much so that the tetraethoxysilane (TEOS)-based compounds are nowadays the most frequently and widely used for preservation in stone heritages [33,34,35].

More recently, an increasing number of studies demonstrated that, in the consolidation processes, the use of particles of nanometric dimensions significantly changes material properties, first in terms of increased surface areas and chemical reactivity. At the same time, it offers positive peculiarities, such as stability, low toxicity and capability to be functionalized with a wide range of molecules and polymers [36,37,38,39].

Among silica-based consolidants, lithium silicate started to be used as a substitute for sodium and potassium silicates almost exclusively in the stabilization of Portland cement concrete, and only scarce recent literature deals with its consolidative abilities in the safeguard of the cultural heritage [40,41]. Nevertheless, it shows several advantages such as the possibility of being applied in wet conditions and overcoming some of the known incompatibility issues encountered with silanes applied to calcite substrates [33,41]. In fact, in presence of water and CO_2_, lithium silicate (n SiO_2_∙Li_2_O) promotes the formation of lithium carbonate, which is able to provide high compatibility with calcium present in the porous structure, improving in this way the consolidation mechanism for lime-based materials which represents a valuable alternative to cement in heritage repair mortars or plasters [42]. Further, unlike most inorganic consolidants that give rise to the formation of soluble salts as reaction by-products (precipitation or chemical reactions with the stone), resulting often in an accelerated surface stone decay, lithium silicate offers the advantage of forming insoluble or poorly soluble lithium salts that scarcely affect the stone surface [41]. Moreover, the lithium silicate supplied as a moderately viscous solution in water provides the undeniable advantage of not being harmful and avoids problems associated with VOC components, which makes the product suitable also for closed and poorly ventilated spaces.

In this research, two inorganic consolidants, a lithium silicate solution and a silica nanoparticle suspension, have been tested for two macroporous volcanic stone materials: Neapolitan Yellow Tuff (NYT) and Campanian Ignimbrite (CI).

In particular, consolidation treatments were carried out using different application methodologies (brushing and full immersion). Then, an extensive characterization was performed in order to evaluate the efficacy of each consolidant compound.

The choice of these stones was due to their relevance in the historical and architectural heritage of the Campanian region (southern Italy). In fact, these zeolitic tuffs were more extensively used locally as materials for construction since Roman times, mainly due to their wide availability, easy workability, chemical–physical features and excellent pozzolanic activity [43,44,45]. However, despite their widespread use, NYT and CI are often characterized by limited performances in terms of durability, primarily due to their high degree of porosity and textural and compositional heterogeneity, which cause a strong requirement of consolidation interventions to prevent and control the unavoidable and significant weathering phenomena [46,47,48] In fact, these volcanic stones are seriously prone to decay leading to gradual stone deterioration as a result of several physical and chemical mechanisms such as moisture infiltration [25,49], salt crystallization [50] and freezing and thawing [51]. So, the performing of consolidation treatments represents an effective methodology to hinder the decay processes, improving the physical–mechanical properties of weathered stones through the reduction in porosity and the increase in surface cohesion [52].

## 2. Experimental

### 2.1. Materials

#### 2.1.1. Natural Stony Materials

Campanian Ignimbrite formation is a volcanoclastic rock widespread over the Campania region (area of about 3000 km^2^) and therefore also the most used as building or ornamental stone [53,54]. The yellow CI unit shows a typical chaotic texture, characterized by assemblages of a yellow ash matrix, dark grey pumiceous scoriae and different authigenic phases, primarily feldspars and zeolites [53,54]. Several samples of yellow lithified deposits of Campanian Ignimbrite were collected in a quarry located in Comiziano (Naples, Italy). The NYT formation is the most important pyroclastic product of Campi Flegrei, and the lithified yellow facies represents the most diffused fair-faced building material in the historical architecture of Naples (Southern Italy) since the Greek–Roman ages [45]. Like CI, the NYT as stone material exhibits high heterogeneity and a huge variability of textural features, resulting from the concomitant occurrence of lithic fragments, pumices, crystals (mainly alkali feldspar, phillipsite and, subordinately, chabazite and analcime) and amorphous phases embedded in a yellow ash matrix [45].

The investigation was carried out on one of the most representative lithofacies of NYT, and samples came from the Edificante quarry in Chiaiano (Naples, Italy).

The characterization of NYT and CI samples was conducted on cubes (4 × 4 × 4 cm^3^) and slabs (5 × 5 × 2 cm^3^) rinsed in distilled water and finally dried in an oven (T = 60 °C) until the achievement of the constant mass.

#### 2.1.2. Consolidant Products

The silica-based consolidant Nano Estel (NE) was supplied by CTS S.r.l. (Altavilla Vicentina, VI, Italy). It is an aqueous colloidal suspension of silica nanoparticles with an average size of 10–20 nm and a 30 wt% content of SiO_2_. Sodium hydroxide (NaOH < 0.5%) was used to stabilize the product, which has an alkaline pH (9.8–10.4). The formation of a silica gel and subsequent consolidation mechanism is the result of water–solvent evaporation that causes the bonding of silica particles.

Lithium silicate solution (LS) (Li_2_SiO_3_, wt%: SiO_2_ 20–25; Li_2_O 2–3) was provided by Prochin Italia S.r.l. (Marcianise, CE, Italy). As described for Nano Estel, silica gel (SiO_2_) is also the desired consolidating product in this case, in addition to a moderate amount of lithium carbonate [41]. In particular, SiO_3_^2-^ becomes silicic acid by hydrolysis, according to the following reaction:

Li_2_SiO_3_ + 3H_2_O => Si(OH)_4_ + 2LiOH

Finally, silica gel is obtained through silicic acid condensation with OH^−^ groups present within stone porosity.

Both consolidants were diluted with demineralized water (1:1) to achieve a better penetration in the stone pores (this issue is further discussed within Section 3.1). The main chemical–physical properties of both consolidants are summarized in Table 1.

#### 2.1.3. Consolidating Treatments

As widely known from the literature, application methodology strongly affects the final results of all consolidating treatments [55,56,57]. Therefore, with the aim of also verifying the specific influence of different kinds of treatment procedures, two different application methodologies were adopted to evaluate the efficacy of the consolidants: brushing and full immersion. The first methodology, which better simulates an onsite consolidation treatment, was used to evaluate the role of the consolidation treatment mainly on the surface of treated lithotypes (i.e., chromatic change, water absorption at low pressure, consolidant adhesion). The second one was used instead to determine the effect of consolidants on bulk sample properties, such as the maximum absorption of consolidant, the compactness and the resistance to salt crystallization. For both the above treatments, consolidants were diluted 1:1 by weight.

The brushing procedure was performed on slab specimens of NYT and CI (5 × 5 × 2 cm^3^). The consolidating products were applied on one surface for each specimen until refusal. Afterward, the specimens were dried at room temperature and then stored in a climatic chamber at 20 °C and HR 50% for 10 days. The samples that underwent the brushing treatment were labeled as NYT/NE_br, CI/NE_br, NYT/LS_br and CI/LS_br.

The consolidating treatments for immersion were carried out on cubic specimens (5 × 5 × 5 cm^3^) of NYT and CI at room temperature (20 ± 5 °C) by soaking the specimens in the consolidant solution for 30 min. The impregnation time was selected based on preliminary tests and was suitable to guarantee the maximum absorption of both consolidants. Then, all treated specimens were stored for 10 days in a climatic chamber (20 °C and 50% RH) to promote the consolidating effect up to complete water evaporation. Finally, all the specimens were weighed until constant mass was achieved, in order to evaluate the average amount of consolidant effectively absorbed. The so-obtained specimens are named throughout the text as NYT/NE_im, CI/NE_im, NYT/LS_im and CI/LS_im.

Moreover, untreated stones, labeled ‘‘NYT/REF” and “CI/REF”, were characterized as reference materials. All the different typologies of samples tested are summarized in Table 2.

### 2.2. Methods

#### 2.2.1. Physical Characterization

Real density (g/cm^3^) was measured by employing a He pycnometer (Micromeritics Multivolume Pycnometer 1305, ±0.1 to 0.2% accuracy) on cylindrical specimens (2.5 cm diameter; height < 3 cm). Open porosity values (%) were subsequently calculated from bulk volume and solid skeletal volume.

Mercury intrusion porosimetry (MIP) was used to conduct a complete pore size analysis (connected porosity, pore size distribution, average pore radius) by means of a Pascal 140/440 Thermo Finnigan apparatus (maximum pressure up to 400 MPa and pore radius analysis up to 0.0019 µm, Thermo Fisher Scientific Inc., Waltham, MA, USA).

The water absorption under vacuum was evaluated according to RILEM Technical Recommendations 25-PEM [58] on cubic specimens (5 cm side) and allowed determining the amount of water uptake (wt%) after full immersion. The samples were dried at 60 ± 5 °C until constant mass (M_1_, g) was reached and were subsequently stored in an evacuation vessel. Then, pressure was lowered to about 20 mm Hg and maintained constant for 24 h. Afterward, tap water at 15–20 °C was gradually introduced into the vessel, maintaining the vacuum condition and keeping it for an additional 24 h. Subsequently, pressure was returned to atmospheric value, and the samples were left underwater for another 24 h and then weighed immersed in water (hydrostatic weighing, *M*_2_, g). Finally, samples were gently wiped with a damp cloth, and the mass of the water-saturated samples (*M*_3_, g) was determined. The water absorption is expressed as follows:

(1) $$\frac{M_{3}-M_{1}}{M_{1}}\times 100$$

**Figure 1:**
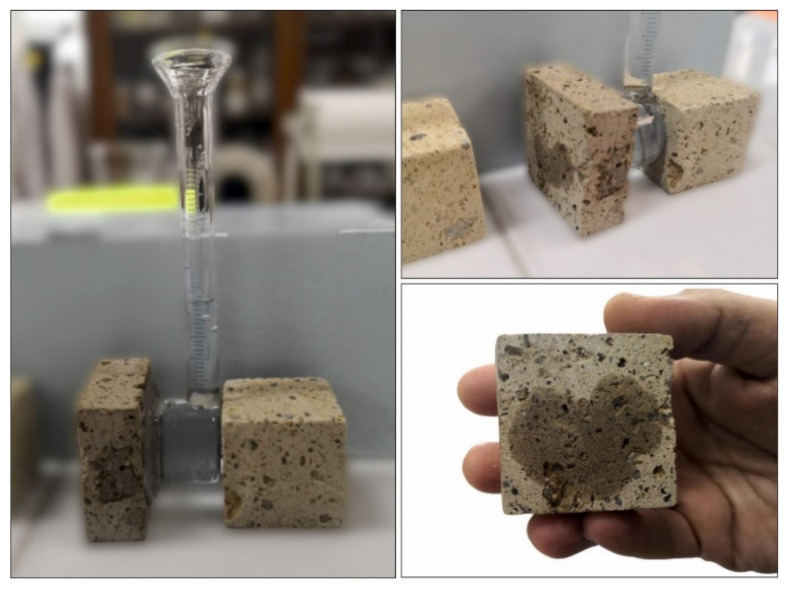
Measurement of water absorption under low pressure by pipe method.

The measurements of water absorption by pipe method were performed on 5 × 5 × 2 slabs, following the suggestions of UNI EN 16302 [59]. This nondestructive method, also adopted for in situ measurements [60,61], involves the use of a Karsten tube to measure the water absorption on the surface of porous inorganic materials under low pressure, so simulating the pouring rain conditions. This test allows estimating the natural stone weathering rate and/or the efficacy of any treatment or aging. The cylinder tube was filled with distilled water (Figure 1), and the test was performed by measuring the volume of water absorbed through a specific stone surface (mL/cm^2^) at scheduled time intervals (min).

Moreover, ultrasonic P-wave velocities were recorded on cubic specimens (4 cm side) according to UNI EN 14579 [62] using a BOVIAR DSP UTD 1004 Ultrasonic device (55 kHz transducers in direct arrangement, BOVIAR srl, Naples, Italy). The nondestructive measurement of P-wave velocities within the stony materials helped to qualitatively estimate both natural stone compactness and the strengthening action of consolidants before and after treatments [57,63,64].

Rheological characterization was primarily carried out using a stress-controlled shear rheometer (Anton Paar MCR 502e, Anton Paar GmbH, Graz, Austria) equipped with DG27 double gap concentric cylinder measurement system for low viscosity. The temperature was fixed at 25 °C. Shear viscosity *η* as a function of the imposed shear rate *𝛾̇* (10^−3^ to 10 s^−1^) was calculated for both NE and LS solutions.

#### 2.2.2. Morphological Characterization

Scanning electron microscopy analyses (SEM) were performed in order to characterize the morphological appearance of stone surfaces before and after the consolidation treatments. All the analyses were carried out using a Cambridge S440 apparatus (acceleration voltage: 20 kV, low vacuum conditions pressure: 10^−5^ mbar).

#### 2.2.3. Chromatic Modifications

Colorimetric tests were performed, according to European Standard UNI EN 15886 [65], in order to verify the surface chromatic changes after treatments. Tests were carried out in triplicate (CM-2500d Konica Minolta spectrophotometer, Konica Minolta sensing Europe B.V., Milan, Italy) at the initial stage on the untreated surface and in a second stage on the same surface of the sample 10 days after the brushing treatment to prevent chromatic changes due to the heterogeneity of the tuff stones. The following parameters were used for the test: 8.0 mm diameter viewing aperture, specular component included (SCI), illuminant D65 and 10° observer angle. The color change (∆E) was determined using the following equation (Equation (2)):

∆E = [(∆L*)^2^ + (∆a*)^2^ + (∆b*)^2^]^1/2^ (2)

where L* is the lightness/darkness coordinate, a* the red/green coordinate and b* the yellow/blue coordinate according to the CIE (Commission Internationale d’Eclairage) [66]. It is important to bear in mind that the surface color variation is generally not perceived by the human eye if ∆E < 3, while when ∆E > 5, an observer can clearly notice two different colors looking at the examined surface [67]. Therefore a ∆E of 5 can be considered as the maximum threshold value generally accepted as a chromatic alteration for stones that undergo consolidating treatments [68].

#### 2.2.4. Peeling Test

**Figure 2:**
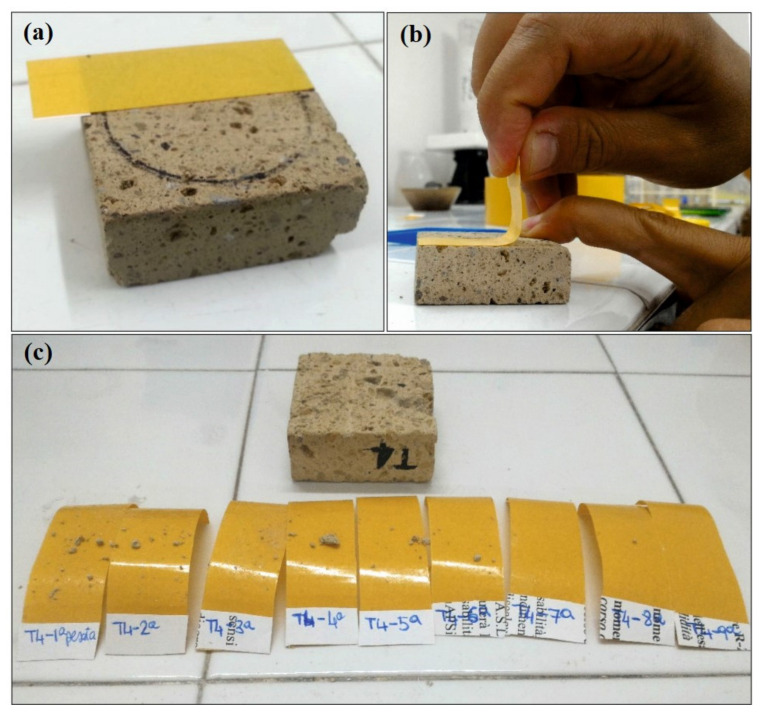
(**a**) Application of tape strips on sample; (**b**) removal, keeping a 90°contact angle; (**c**) tape strips after tear.

The peeling test, also known as “Scotch Tape test method”, is a quick and easy surface test to estimate the adhesion of a coating layer to a substrate [69,70]. It has been used since the 1960s for evaluating the surface cohesion qualities of historic building materials [71], even if there are not any standards or reliably verified recommendations in support of its application in the cultural heritage conservation field [69]. Experimental tests were performed in triplicate on slab specimens (5 × 5 cm^2^) before and after the brushing procedure using double-sided tape (Sicad Group, strips of 2 × 5.5 cm). Tape strips were stuck and left on samples for 60 s and then removed, keeping a 90°contact angle between the sample surface and tape (see Figure 2). Each tape strip was weighed before and after the test in order to determine the detachment of stony powder and/or fragments after tearing. The peeling test was repeated several times on the same surface area until weight variations became negligible.

#### 2.2.5. Determination of Resistance to Salt Crystallization

The resistance of stone to salt crystallization was evaluated following the procedure described in the standard UNI EN 12370:2001 [72], which is adopted for stones with a porosity greater than 5%. These achievements, frequently coupled with other accelerated aging tests (e.g., cyclic freezing–thawing) are widely used methods to estimate the extent of damage suffered by stony materials when exposed to decay agents and the general durability of natural stones [73,74,75,76]. In particular, salts growing in porous hosts represent a major source of decay for natural building stones; therefore, a lot of research has been devoted to this topic [50,77,78,79,80,81,82]. In this case, six cubic specimens (4 cm side) of each volcanic lithotype (both untreated and treated by full immersion) were stored in a climatic chamber at T = 20 °C and Hr = 50% until constant weight (*M_0_*) and then immersed in a solution of sodium sulfate decahydrate (C = 14% wt./wt.) for 2 h at T = 20 °C. Subsequently, the specimens were progressively heated in 10 h to 105 °C and stored at this temperature for 16 h. Finally, they were cooled at room temperature, stored for 1 day in the climatic chamber (20 °C, 50% Hr) and weighed (*M_i_*). The above-mentioned cycle was repeated until disaggregation of the specimens or for a maximum of 15 times.

After every cycle “*i*”, the mass variation (*m_v_*) was expressed by means of the ratio between the weight after “*i*” cycles (*m_i_*) and the initial weight (*m_0_*):

(3) $$m_{v}=\frac{m_{i}}{m_{0}}$$

The results of the test were expressed by means of the above mass variation, by the number of cycles necessary to obtain the disaggregation of the specimens and by a photographic report.

## 3. Results and Discussion

### 3.1. Consolidant Absorption

Weight increases (wt%) of the NYT and CI specimens after full immersion in the consolidant solutions are given in Table 3. The values reported are the average of three measurements for each consolidated lithotype.

It is immediately possible to argue that the consolidant agents can penetrate more easily into the stone pore network of NYT rather than CI. Above all, this remark can be explained taking into consideration that NYT is characterized by a higher porosity compared to CI (see Table 4).

**Figure 3:**
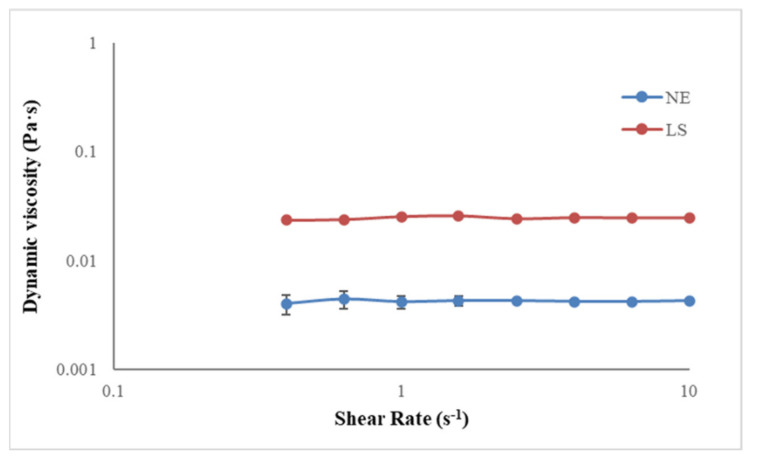
Dynamic viscosity test for NE and LS consolidants.

Moreover, it is possible to notice that a higher amount (wt %) of NE water suspension penetrates NYT and CI specimens compared to LS solution. This evidence could be related to the different viscosity of the two consolidating agents as confirmed by the results of the viscosity test reported in Figure 3.

Both the consolidants showed the rheological behavior typically related to Newtonian fluids, because their viscosity is constant and not affected by the variation of the shear rate. In particular, the average values of the dynamic viscosity obtained are 0.004 Pa∙s and 0.025 Pa∙s for NE and LS respectively. The higher value of LS viscosity, compared to that of NE, could explain the lower amounts of consolidant penetrated into both the treated stony materials.

After 10 days of curing and the evaporation of solvent (water) from solution, the silicate hydrolysis–condensation reactions occurred for both consolidant products. At that stage, the weight increase related to NE treatment was about 5.6% for NYT and 3.0% for CI, while the weight increase related to LS treatment resulted to be about 5.0% for NYT and 2.4% for CI. These values represent the actual amount of silica gel deposited within the voids and pores after the curing period.

### 3.2. Physical Characterization

The effects of consolidant treatments on some relevant physical characteristics of volcanic stones (i.e., density, open porosity and water absorption) were evaluated, and the experimental results are listed in Table 4.

Firstly, it should be noted that there was a moderate increase in apparent density for NYT and CI specimens as a consequence of LS and NE treatments. Further, NYT showed values of open porosity (60.65%) and water absorption (53.12%) significantly higher than those of CI (50.20% and 38.57%, respectively). This difference in terms of porosity accessible to water justifies the higher amounts of both consolidants that entered into the pore network of NYT compared to CI (see Table 3). In general, both NE and LS treatments resulted in a slight decrease in the percentage of the open porosity of volcanic stones (2–3%), but consolidants were responsible for greater efficiency in reducing the water absorption capacity of NYT and CI (up to 9%). In fact, the water uptake/movement properties are drastically influenced by consolidation, mainly due to alteration in pore geometry [83,84].

**Figure 4:**
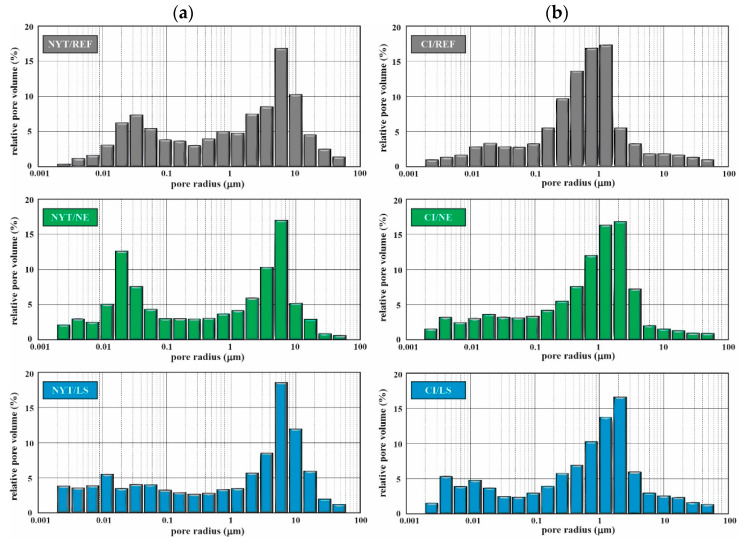
Pore radius distribution of NYT (**a**) and CI (**b**) samples, untreated (REF) and treated with Nano Estel (NE) and lithium silicate (LS).

MIP analyses were carried out on the surface portion of tuff specimens (up to 5 mm) in order to examine the changes in the porous network caused by consolidant impregnation (Table 5, Figure 4). Hg porosimetry results confirmed the overall decrease in connected porosity after consolidant application. In particular, NYT/REF pattern exhibited a known bimodal distribution of pores [59], with a first frequency peak in the macropore range (from 1 to 10 µm; average pore radius equal to 6.81 µm) and a second smaller one right above the meso-macropore domain (from 0.01 to 0.1 µm). NYT/NE representative histogram plot showed, on the whole, the same distribution of untreated reference but was slightly shifted towards smaller size pores. Quite different is the distribution of NYT/LS, with the almost complete drop of the second peak resulting in an overall flattening distribution up to 2 µm. It should be underlined that conservative treatments (NE and LS) altered the porous space, especially in NYT/LS where the frequency of pores in the 0.01–0.1 μm was so much decreased that the water rate absorption was significantly influenced.

Further, the unimodal pore-size distribution of CI/REF evidenced the predominance of macropores (most of the pore volumes range between 0.1 and 2 µm, with an average pore radius occurring at 1.13 µm); pore-size distribution of treated samples (CI/NE, CI/LS) showed in general very similar trends, except for a narrowing of principal peak distribution clearly trending towards coarser pores (average pore radius was about 2 µm).

**Figure 5:**
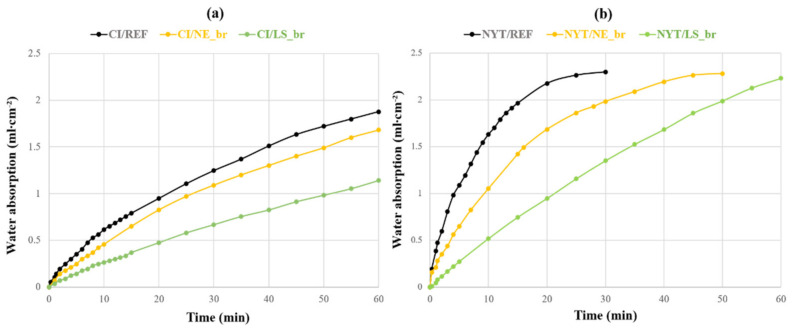
Water absorption by pipe method for (**a**) reference and treated CI samples and (**b**) reference and treated NTY samples.

Since consolidation treatments mainly affected the pores of the surface layers, water absorption kinetics was assessed by the water absorption at low pressure both on the reference and brushed samples (Figure 5). The water absorption curves of CI (Figure 5a) unequivocally show a marked difference in water uptake of the two treated stones; CI/LS_br path revealed a lower slope than untreated sample; consequently, this implies that LS consolidant significantly changed and decreased the low-pressure water permeability on the CI surface, also reducing the water absorption rate. Furthermore, CI/NE_br curve displays a similar trend during the test, but it results in being less effective to reduce the water absorption if compared with CI/LS_br. As regards NYT curves (Figure 5b), both treatments significantly changed the stone permeability, lowering the amount of water absorbed by the treated surfaces. In particular, there was a noticeably good performance exhibited by the NYT/LS_br specimen, which did not reach complete water saturation before the end of the test unlike NYT/REF and NYT/NE_br specimens (after 30 and 50 min, respectively).

The propagation of ultrasonic P-waves in stony materials represents a significant parameter useful to assess the efficacy of consolidation treatments [85,86]. Consolidation, in fact, should improve the stone mechanical properties, giving cohesion to the weathered parts and increasing compactness [63]. The ultrasonic pulse velocity (UPV) measurements show that both consolidation treatments led to an increase in the compactness of all tuff samples, deducible from the increase in corresponding UPV values. In particular, CI samples showed an increase in UPV values equal to about 5% and 12% for NE and LS, respectively. UPV increases for NYT resulted in being more similar for both treatments (8% for NE and 10% for LS) (Table 6). In general, these considerations further confirm that LS resulted as being the more effective consolidant for both volcanic stony materials

### 3.3. Morphological Characterization

**Figure 6:**
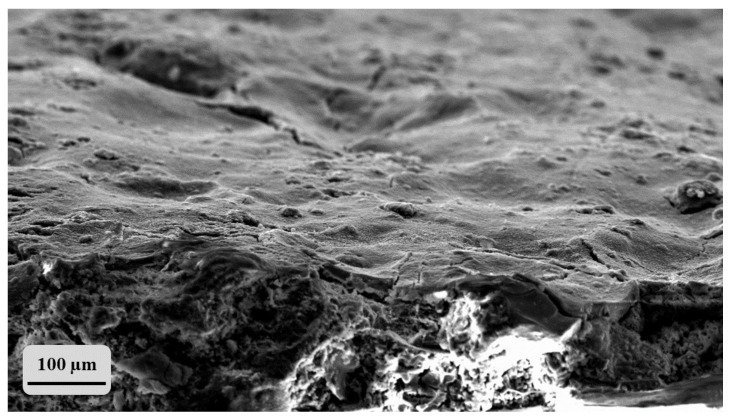
SEM image of CI after brushing treatment with pure LS.

**Figure 7:**
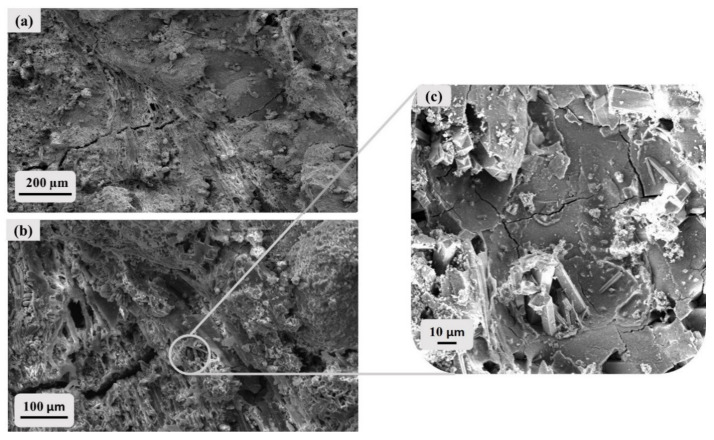
SEM images of NYT and CI at different magnifications after brushing treatment with LS solution (dilution 1:1): (**a**) CI (100X), (**b**) NYT (400X), (**c**) NYT (1000X).

SEM observations on the surface of treated specimens by brushing procedures are reported in Figure 6 and Figure 7.

As an example, the micrograph of CI surface treated with pure lithium silicate (Figure 6) clearly shows that the surface is covered by a uniform and compact layer of consolidant, which probably inhibits further consolidating agent from entering into the pores, as also confirmed by previous research concerning NE treatments of tuff stones [52].

Therefore, in order to improve the penetration into the porous pattern of stones, both consolidants were diluted (1:1) and as a consequence, in some instances, the surface textures of glass shards and pumice fragments of both CI and NYT are still recognizable (Figure 7a,b). At higher magnification, the consolidating agent is clearly visible, as are the crystalline grains that seem to be wrapped in a gel layer (Figure 7c).

### 3.4. Chromatic Modifications

The measurements performed with the color spectrophotometer, concerning the unavoidable and undesirable chromatic changes caused by the brushing treatments, are listed in Table 7.

**Figure 8:**
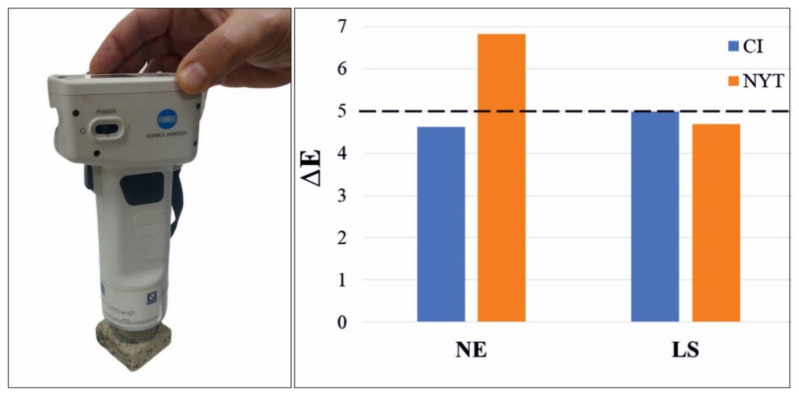
Spectrophotometer for chromatic measurements (**left**) and total color changes (ΔE) for all the brushing treated samples (**right**).

It is worth observing that more samples belonging to the same stone lithotypes may exhibit a different chromatic aspect, due to the wide heterogeneity of the above stones. So for each treatment, a different untreated stone was used as chromatic reference (listed as REF1 and REF2 in Table 7). Firstly, it is possible to note that all the consolidating treatments induced a certain degree of darkening and yellowing of the treated surfaces. In particular, after brushing treatment with LS, the “L” color coordinate decreased by about 7% for both CI and NYT, while it decreased by about 5.5% and 8% for CI and NYT, respectively, after NE treatment. This evidence leads to the conclusion that NE more significantly affected the visual chromatic appearance of tuff samples than LS. As further confirmation, the total color differences (ΔE) of all the treated tuff samples are reported in Figure 8, which clearly shows that the color variation promoted by LS is similar when the product is applied on both CI and NYT, while the color variation promoted by NE differs substantially for the two lithotypes considered.

Moreover, the whole color changes were ΔE > 3, thus meaning that the color variations related to all consolidating procedures performed are above the human eye detection limit, independently from the type of consolidant used [85]. Anyway, LS treatments gave rise to lower perceptible chromatic variations on both treated stone surfaces (ΔE < 5) after stone consolidating treatments [67]. This is not true for NYT/NE_br samples, which showed ΔE > 5 (Table 7).

It is necessary, however, to clarify that the stone chromatic changes can be probably related to the application procedure used for their consolidation. In fact, even if all treatments tend to have some degree of influence in terms of color impact, brushing treatments resulted in general in higher color variations, having a greater impact on color than the immersion and capillary treatments, whatever the stone type [87].

**Figure 9:**
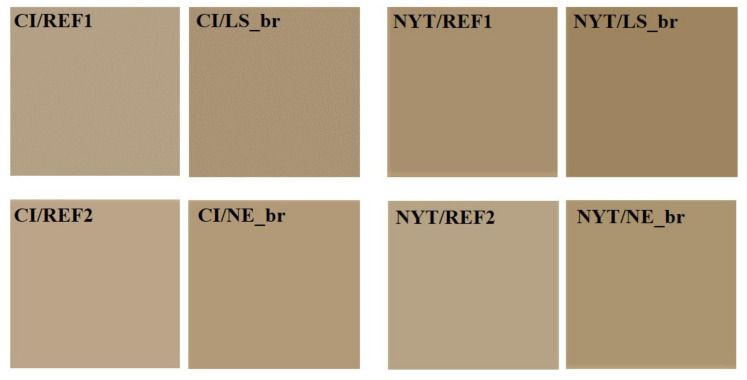
Chromatic variations of untreated and treated CI and NYT samples.

Moreover, in order to obtain a representative image of the color variations induced on treated tuff samples, the EasyRGB website [88] on was used to convert graphically the L *, a * and b* coordinates, and the results obtained are reported in Figure 9.

### 3.5. Peeling Test Results

**Figure 10:**
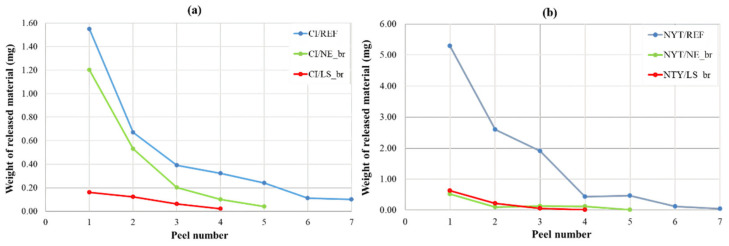
Scotch tape test results on CI (**a**) and NYT (**b**) samples before and after brushing treatment with NE and LS.

Scotch tape tests were performed by weighing the amount of powder and/or fragment material stuck to the adhesive tape that was repeatedly attached to and removed from the stones, in order to estimate the surface cohesion of CI and NYT before and after consolidating treatments [69]. The experimental tests were carried out for each sample until no weight change was recorded, so the maximum peel number was different for all the tested samples. The results, reported in Figure 10, showed a progressive decrease in the removed material after every tear, mainly marked for untreated samples of NYT and CI. Further, the good efficiency of surface treatments appears evident, especially for NYT samples, which exhibit a clear superficial reinforcement effect if compared with untreated samples. In fact, both consolidants led to a significant reduction (about 90%) of material loss from the first tear (Figure 10b), and the amount of released material started to be almost constant from the second removal of tape. Similar behavior can be noticed for CI/LS_br, while a different trend can be identified for the CI/NE_br sample, which showed a lower material loss reduction equal to about 23% (Figure 10a) after the first tear. This represents a further confirmation of the higher efficacy of the LS treatment when performed on CI supports.

### 3.6. Salt Crystallization Resistance

The crystallization of soluble salts represents one of the crucial mechanisms of stone deterioration, and the accelerated aging test, performed with sodium sulfate solution, accordingly induces a strong degradation of porous stones. In fact, as already fully reported in previous studies [48,89], a severe failure was recorded after this accelerated test for both macroporous stones, which are in general totally disaggregated prior to completion of the required cycles.

**Figure 11:**
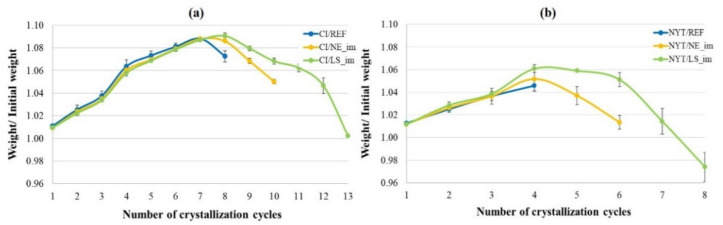
Salt crystallization resistance results of CI (**a**) and NYT (**b**) after aging test.

**Figure 12:**
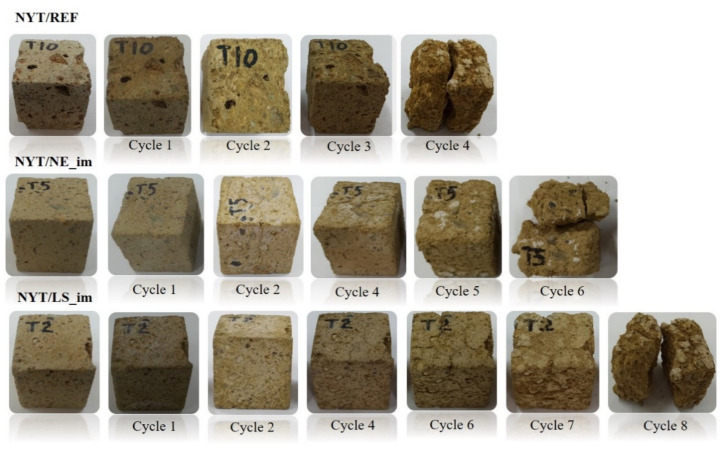
NYT samples during salt crystallization test.

**Figure 13:**
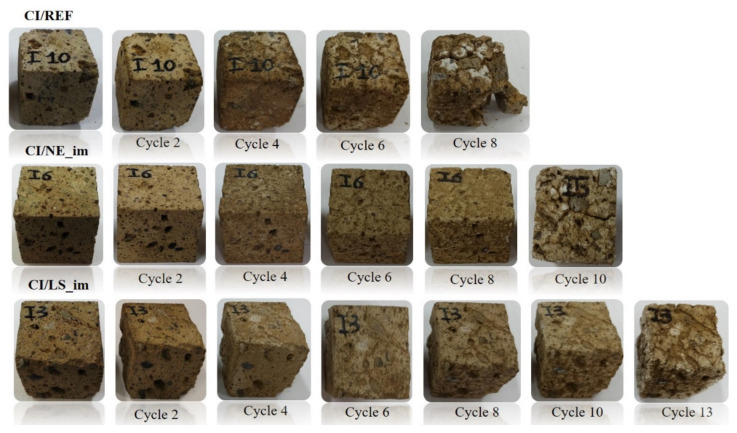
CI samples during salt crystallization test.

The salt damage of stones is commonly expressed as their weight loss (Figure 11), and the stone specimens were routinely visually inspected after cyclic crystallization (Figure 12 and Figure 13).

Overall, it is worth observing that all curves of Figure 11 exhibit a first increasing step, where the mass increase is the main phenomenon due to the salt absorption, followed by a decreasing step, where instead the loss of material, due to sample disaggregation, represents the prevalent mechanism. By observing these graphs, it appears evident that CI samples show higher resistance to salt crystallization than NYT samples [48]. Moreover, for both lithotypes, the consolidating treatments strongly enhanced the number of cycles after which the complete disaggregation of the specimens occurs. In particular, in accordance with the previous results, LS seems to lead to the best performances because the resistance to crystallization cycles almost doubles for both lithotypes, going from 4 to 8 for NYT and from 8 to 13 for CI.

Visual examination revealed that untreated NYT specimens that underwent salt crystallization test rapidly presented damage (Figure 12), and visible effects (pulverization, rounding edges, etc.) appeared during the first cycles, up to complete disintegration after four cycles. Both the treated samples appeared to be still compact after four cycles and showed evident fracturing and loss of material after five or six cycles; total breakdown was observed after six and eight cycles for NYT/NE_im and NYT/LS_im, respectively (Figure 11).

The photographic report of CI specimens (Figure 13) highlighted a better resistance of CI to salt crystallization if compared with NYT. In fact, a marked rounding of the edges and a continuous whitish patina (efflorescence) of untreated CI specimens are visible effects starting from four cycles; then, CI breakage occurs after eight cycles. Both consolidated specimens did not undergo any severe damage excluding a minor pulverization until 4 cycles, then a progressive exfoliation affected treated CI starting from 4 to 8 cycles (10 for CI/LS_im); finally, at 10 (CI/NE_im) and 13 (CI/LS_im) cycles, the failure appeared as deep cracks that compromised sample integrity.

## 4. Conclusions

The strategies for the conservation of historic stone buildings and monuments have been widely debated by the research community for a long time. In the modern era, the heightened focus of ‘conservation scientists’ is also due to the aggravating circumstance of increasing global air pollution and climate change and the need to find innovative sustainable solutions. In fact, in order to avoid all limitations and drawbacks of traditional consolidants, in recent years, new approaches have been experimented with in terms of sustainability by using environmentally friendly products for stone consolidation. This research focused on two volcanic tuffs, Neapolitan Yellow Tuff and Campanian Ignimbrite, which suffering severe deterioration in urban areas due to their textural and compositional heterogeneity and high porosity, especially when exposed to aggressive weathering agents such as soluble salts. The consolidation treatments were carried out by pursuing major objectives such as eco-friendly solvent products (i.e., water), chemical compatibility between consolidants and siliceous volcanic stones, preservation of aesthetic features and durability.

Two different consolidant compounds that fulfilled these requirements were tested on volcanic stones: a silica nanoparticle dispersion and a lithium silicate solution.

Two different application procedures of consolidants were chosen: brushing and total immersion. The treatment by brushing (similar to the common on-site practice of consolidation) was chosen to assess the modification of stone surfaces by measurements such as water absorption (by pipe method), adhesion/cohesion (peeling test) and color (spectrophotometry). The full immersion method was carried out in order to test the variation of main physical properties (density, accessible porosity, Hg porosimetry, water absorption), to estimate the strengthening action of consolidants (UPV velocities) and to assess the durability of treated tuff stones (salt resistance).

Laboratory data confirmed the effectiveness of the consolidation treatments, especially in terms of lowering porosity and pore accessibility to water/moisture, moderately improving the mechanical resistance and not affecting the visual aesthetic of stone surfaces.

In particular, lithium silicate yielded promising results and usually exhibited better performance, despite the lower amount of silica deposited into the pores.

In fact, this consolidating agent consistently enhanced the superficial cohesion of volcanic stones and was more efficient in reducing the surface water absorption. Further, the aging tests have provided remarkable results if compared with the untreated stones, exhibiting an extreme resistance to salt crystallization, for a number of cycles even double, in the case of NYT.

In conclusion, it is possible to propose the use of lithium silicate as a consolidant agent for the restoration of all the load-bearing masonry and cladding structures manufactured with NYT and CI, in particular for the historical buildings because of the high compatibility with lime-based mortars.

## Figures and Tables

**Table 1:** Main properties of the two consolidants used.

	Nano Estel	Lithium Silicate
Supplier	CTS S.r.l	Prochin Italia S.r.l.
Physical state	Liquid	Liquid
Color	Colorless/Transparent	Colorless/Transparent
Content of SiO_2_ (wt%)	30	20–25
Specific weight (g/cm^3^ at 20 °C)	1.2	1.19–1.21
Content of Li_2_O (wt%)	/	2–3
Specific surface (m^2^/g)	260	/
pH	9.5-10.4	10.5–10.8
Particle size	10–20 nm	/

**Table 2:** Schematic summary of all the different samples tested.

Sample Name	Stone Support	Consolidant Agent	Consolidation Procedure
CI/REF	Campanian Ignimbrite	/	/
NYT/REF	Neapolitan Yellow Tuff	/	/
CI/NE_im	Campanian Ignimbrite	Nano Estel	Immersion
NYT/NE_im	Neapolitan Yellow Tuff	Nano Estel	Immersion
CI/LS_im	Campanian Ignimbrite	Lithium Silicate	Immersion
NYT/LS_im	Neapolitan Yellow Tuff	Lithium Silicate	Immersion
CI/NE_br	Campanian Ignimbrite	Nano Estel	Brushing
NYT/NE_br	Neapolitan Yellow Tuff	Nano Estel	Brushing
CI/LS_br	Campanian Ignimbrite	Lithium Silicate	Brushing
NYT/LS_br	Neapolitan Yellow Tuff	Lithium Silicate	Brushing

**Table 3:** NE and LS consolidant absorption after full immersion treatments (average values ± standard deviation).

Consolidant Absorption (wt %)		
	After Full Immersion for 30 min	After 10 Days Curing (20 °C, 50% HR)
NYT/NE_im	36.97 ± 0.22	5.63 ± 0.50
CI/NE_im	22.42 ± 0.21	3.04 ± 0.09
NYT/LS_im	29.66 ± 0.28	5.02 ± 0.16
CI/LS_im	12.75 ± 0.84	2.40 ± 0.14

**Table 4:** Main physical properties of tuff stones before and after consolidating treatments, carried out on at least six samples (average values ± standard deviation are reported).

Sample	Apparent Density (g/cm^3^)	Real Density (g/cm^3^)	Open Porosity (%)	Water Absorption (%)
CI/REF	1.16 ± 0.05	2.30 ± 0.03	50.20 ± 2.29	38.57 ± 1.01
NYT/REF	1.02 ± 0.05	2.40 ± 0.04	60.65 ± 2.07	53.12 ± 0.97
CI/NE_im	1.20 ± 0.03	2.28 ± 0.04	47.48 ± 1.03	29.43 ± 0.96
CI/LS_im	1.21 ± 0.04	2.27 ± 0.03	47.65 ± 1.67	29.31 ± 0.61
NYT/NE_im	1.06 ± 0.03	2.37 ± 0.05	57.59 ± 1.69	48.82 ± 1.53
NYT/LS_im	1.07 ± 0.03	2.36 ± 0.04	57.85 ± 1.06	44.23 ± 0.77

**Table 5:** Porosity data of treated and untreated tuff specimens measured by mercury intrusion porosimetry (MIP).

	Total Specific Surface Area (m^2^/g)	Average Pore Radius (μm)	Total Porosity (%)
NYT/REF	13	6.8	49.0
NYT/NE_im	27	5.6	44.2
NYT/LS_im	19	7.1	45.3
CI/REF	14	1.1	45.2
CI/NE_im	20	2.0	41.8
CI/LS_im	16	1.9	42.1

**Table 6:** Ultrasonic pulse velocity (UPV) for CI and NYT samples before and after consolidating treatments by full immersion.

Sample	UPV (m/s)
CI/REF	1733.00 ± 20.26
NYT/REF	1666.21 ± 21.11
CI/NE_im	1820.06 ± 38.88
CI/LS_im	1932.24 ± 29.22
NYT/NE_im	1794.22 ± 45.32
NYT/LS_im	1831.91 ± 44.82

**Table 7:** Chromatic modifications of tuff stones after brushing treatment. L*: black-white color; a*: red-green color; b*: yellow-blue color; ∆E: color change.

Samples	L*	a*	b*	∆E
NYT/REF1	60.98	4.08	21.06	4.69
NYT/LS_br	56.87	4.53	23.24	
NYT/REF2	68.03	2.02	18.26	6.83
NYT/NE_br	62.57	2.33	22.30	
CI/REF1	67.05	2.65	17.02	4.98
CI/LS_br	62.46	3.43	19.11	
CI/REF2	68.66	4.03	17.96	4.62
CI/NE_br	64.85	4.42	20.54	

## Data Availability

The data presented in this study are available on request from the corresponding author.
